# Brain mechanisms of oral multisensory processing related to oral health: a systematic review of neuroimaging findings

**DOI:** 10.1038/s41405-025-00339-3

**Published:** 2025-05-14

**Authors:** Chia-Shu Lin, Shih-Yun Wu

**Affiliations:** 1 Department of Dentistry, College of Dentistry, National Yang Ming Chiao Tung University, Taipei, Taiwan; 2 Institute of Brain Science, School of Medicine, National Yang Ming Chiao Tung University, Taipei, Taiwan; 3 Brain Research Center, National Yang Ming Chiao Tung University, Taipei, Taiwan; 4 Oral Medicine and Innovation Center, National Yang Ming Chiao Tung University, Taipei, Taiwan; 5 Department of Stomatology, Taipei Veterans General Hospital, Taipei, Taiwan

**Keywords:** Dentistry, Oral diseases

## Abstract

**Aim:**

Oral functions related to eating, including mastication, swallowing, and taste, are fundamentally a multisensory experience that relies on the crossmodal interaction of touch, gustation, temperature, pain, and proprioception. The brain mechanisms of oral multisensory processing related to eating have remained unknown.

**Methods:**

The current systematic review summarizes the findings from neuroimaging studies (mainly functional magnetic resonance imaging) focusing on the interaction of multiple sensory stimuli in human participants. Neuroimaging studies of human adults on the interaction between multiple sensory stimuli related to oral functions were identified and extracted via three electronic databases and reviewed according to the Preferred Reporting Items for Systematic Reviews and Meta-Analyses.

**Results:**

Thirteen primary studies were eligible to be included in this review. Five studies investigated the interaction of intraoral (i.e., sensorimotor, taste, and noxious) stimuli. Six studies investigated the interaction between intraoral and extraoral (i.e., auditory, olfactory, and visual) stimuli. One study investigated the audio-visual interaction on dental fear, and another study investigated sensorimotor processing of eating tools. The studies showed great diversity in the experimental design of crossmodal interaction. Regarding the brain features related to the interaction, the somatosensory and motor regions were mostly reported in the studies.

**Conclusions:**

The systematic review revealed a complex pattern of brain activation of oral multisensory processing, which can be attributed to the diversity in the experimental design of crossmodal interaction. The findings highlight the role of multisensory integration in maintaining oral health.

## Introduction

Our perception of the inner state of the oral cavity is fundamentally a multisensory experience. The experience of a specific sensory modality is formed by pathways of primary sensation. For example, the experience of the texture and taste of food is shaped by the tactile and the gustatory pathways, via mechanoreceptors and chemoreceptors, respectively. Moreover, sensory processing of these primary stimuli is integrated to form a holistic experience of food intake. For example, the somatosensation of texture (i.e., light touch) and pressure (i.e., deep touch) is integrated into oral stereognosis, a somatoperception about the perception of intraoral objects [1]. During chewing, the sound of food (e.g., crispy or crunching) may affect our perception of its flavor [2]. The viscosity of food, which relates to the kinesthesis of the masticatory system, may be associated with the perception of its taste [3]. Furthermore, the crosstalk between multiple sensory experiences may affect patients’ experience of dental treatment. For example, the sensation of pain, the sound of the drill, and the presence of the needle are all associated with individual dental anxiety [4]. Until now, the role of multisensory processing in dental practice and oral health has remained unclear.

Multisensory integration refers to ‘the neural integration of different sensory modalities’ [5]. The integration further gives rise to ‘changes in behavior associated with the perception of and reaction to those stimuli’ [6]. Therefore, multisensory processing of sensory information is associated with the interaction of multiple unimodal sensations. Such a crossmodal interaction has been widely investigated in neuroimaging research. For example, when individuals received unimodal auditory and visual stimuli, there was corresponding brain activation at the auditory and visual cortices, respectively. When both stimuli, such as auditory speech and visual texts, were given with congruent meanings, there was greater brain activation in the superior temporal gyrus (STG), compared to the brain activation of unimodal stimuli [7]. The findings revealed that the STG participates in a crossmodal (i.e., auditory and visual) interaction or ‘binding’ between these two sensory modalities. At present, neuroimaging studies have revealed that the superior and middle temporal gyri, the thalamus, the insula, and the inferior frontal gyrus may be associated with multisensory integration [8].

In terms of oral neuroscience, there have been review studies on the brain mechanisms associated with a specific part of oral function, including acute dental pain [9], chronic orofacial pain [10], mastication [11], swallowing [12], taste [13], and dental anxiety and fear [14]. However, the issue of multisensory integration in oral functions has been largely ignored, except for research on speech [15]. To bridge this gap, the current review aims to investigate the brain mechanisms of multisensory processing related to oral health, by systematically reviewing the neuroimaging studies on the interaction of multiple sensory modalities in healthy adults. This study focuses on the following three aims:

1. Investigating the brain mechanisms associated with oral multisensory processing, as reported by previous neuroimaging studies.
2. Analyzing the experimental paradigms of crossmodal stimulation related to oral function.
3. Elaborating the translational application of neuroimaging findings on clinical practice.

## Methods

### Eligibility criteria

The eligibility criteria for including studies were defined according to the following features, based on the guideline of the Preferred Reporting Items for Systematic Reviews and Meta-Analyses (PRISMA) [16]:

1. Participants: This review included studies that investigated healthy human subjects. The primary studies that investigated only patients with a disease were excluded.
2. Intervention/Comparison: The review included both observation research (without an experimental intervention) and experimental research. The studies should include at least one condition of oral stimulation (e.g., tactile or taste stimulus) or non-oral stimulation associated with dental treatment of oral health (e.g., a visual scene of dental treatment). Because the current study only focuses on multisensory integration related to oral health, the studies focusing on speech processing were excluded. If the study compares a patient group and healthy controls, only the findings of healthy controls will be focused on in the review.
3. Outcomes: The review focused on the brain features identified by the neuroimaging investigation, including magnetic resonance imaging (MRI), magnetoencephalography (MEG), transcranial magnetic stimulation (TMS), and functional near-infrared spectroscopy (fNIRS). Because we focused on synthesizing the spatial pattern of brain activation across the studies (see Data synthesis), the studies that only reported temporal features of brain activity (e.g., electroencephalography research) or those that did not report the brain regions of activation were excluded. Structural (e.g., grey matter volume) or functional (e.g., brain activation during task conditions) features should be reported in the studies. The studies should report at least two different unimodal stimuli. The studies need to include (a) at least one of the sensory modalities that are associated with oral sensorimotor processing, such as tactile, taste, and mastication, which relates to proprioception and sensory feedback, or (b) non-oral stimuli that are associated with the behavior of food selection or dental treatment (e.g., dental anxiety and fear). Notably, a typical study of crossmodal interaction may include a ‘bimodal’ condition, i.e., the condition when both unimodal are concurrently applied [17]. In this review, the studies either with or without a bimodal condition will be included.
4. Design: Randomized or non-randomized research.

### Search strategies and information sources

Three electronic databases/indices were searched with the eligibility criteria: PubMed, Embase, and Web of Science, with the combination of keywords consisting of neuroimaging, oral function, and multisensory processing (Table 1). The search was conducted from the data of database inception to 2025.1.26. Grey literature (e.g., government reports or conference papers) was not included. No limitation was set on the language of publications.

**Table 1:** Strategy of keyword search for PubMed (by 2025.1.26)

Search number	Query	Results
1	neuroimaging OR MRI OR “positron emission tomography”[mesh] OR PET[tiab] OR “magnetoencephalography”[mesh] OR MEG[tiab] OR “functional near-infrared spectroscopy” OR fNIRS[tiab]	1099572
2	multisensory OR multi-sensory OR crossmodal* OR cross-modal*	14404
3	tooth OR teeth OR dental OR oral OR orofacial OR intraoral OR mouth OR jaw OR tongue	1874450
4	#1 AND #2 AND #3	86

### Selection process

**Fig. 1:**
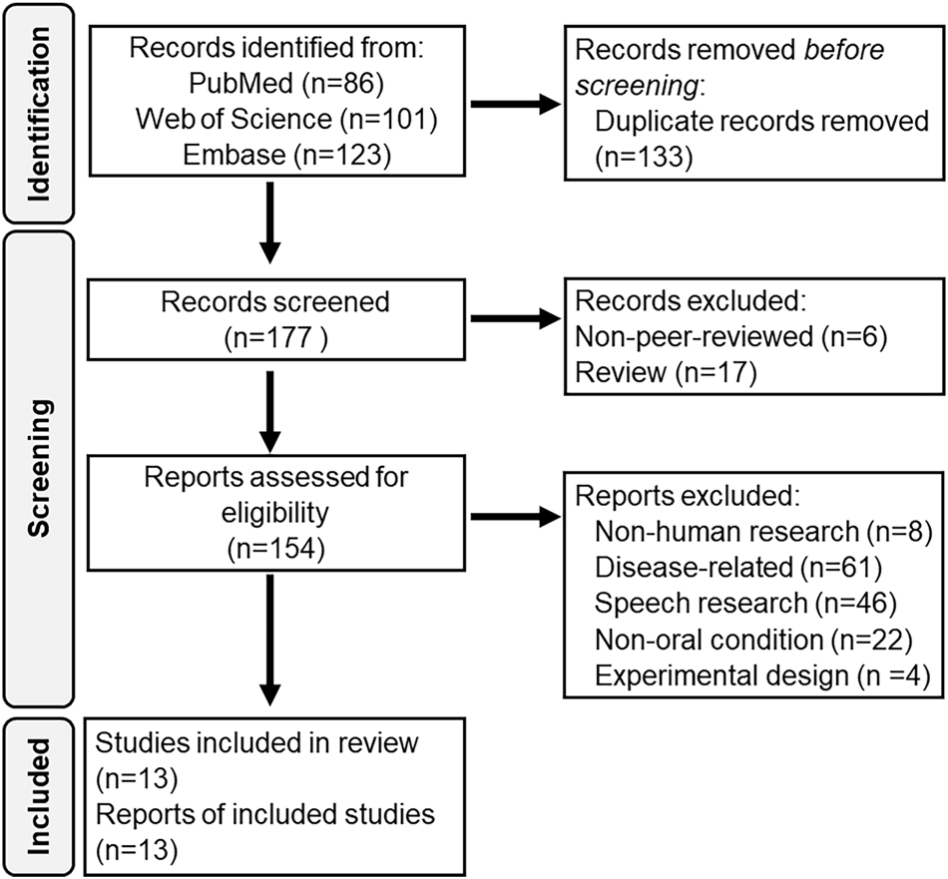
Flow diagram of the systematic review. Flow diagram of searching and screening of eligible articles.

The literature search was conducted independently by two authors (C-S Lin and S-Y Wu). The authors independently screened the titles and abstracts of all the records, after the removal of duplication, according to the eligibility criteria. Within the 177 articles (Fig. 1), the authors reached a high inter-rater agreement in the inclusion of articles (kappa = 0.68). A consensus of the final inclusion of the articles was reached by further discussion between the authors.

### Data collection

The following seven items of study features were extracted from the full text of the included articles by the author: (1) the source of the study, including the name of the first author and the year of publication, (2) the number of participants, including the number for each subgroup based on the study design, (3) the range or mean ± standard deviation of the participant’s age, (4) the method of neuroimaging, (5) the condition of unimodal sensory stimuli, (6) the condition of bimodal stimuli, and (7) the approach to analyze crossmodal interaction. The information for a data item was labeled ‘not available’ (n.a.) if it cannot be identified in a study.

### Data synthesis

Neuroimaging studies focused on the spatial pattern of brain activation, i.e., the results of ‘brain mapping’. In contrast to the analyses of clinical findings (e.g., the effect of pharmacological intervention), data synthesis of neuroimaging research focuses on a consistent pattern of brain activation across the primary studies [18], rather than the pooled effect size (e.g., therapeutic effect) estimated from individual studies. In the current review, the pattern of brain activation associated with oral multisensory integration was synthesized by the labeled-based approach [18]. The brain regions reported in individual studies were first identified from the tables and figures of each study. Notably, different studies may report the brain regions of significant activation with labels from different brain atlases. To reconcile the heterogeneity in labeling brain regions, we re-surveyed the coordinates of loci reported in individual studies systematically using the Automated Anatomical Labelling (AAL) atlas 3 [19]. Subsequently, the findings from all the studies were summarized by tabulation. The hemisphere (left, right, or bilateral) of the brain regions was identified. Notably, because the included studies diverged greatly in the research variables (i.e., sensory modalities) and study design, we did not conduct a coordinate-based meta-analysis due to the great between-study heterogeneity.

### Study risk of bias assessment

The assessment of the risk of bias (RoB) was based on the design of the Newcastle-Ottawa scale (NOS) for cohort studies [20]. The original items were customized for evaluating the RoB of neuroimaging studies, including the following five items: (1) the systemic condition and (2) the oral condition of participants, which were related to the bias of selection of NOS, (3) the design of task comparison (e.g., if a proper control condition was used), which was related to the bias of comparability of NOS, and (4) the correction of head motion and (5) thresholding of imaging results, which were related to the bias of outcome of NOS. Each item was assessed as high or low risk, or uncertain.

### Reporting bias assessment

The reporting bias associated with a missing result, which can be derived from the decision not to publish non-significant findings, may cause a biased conclusion in systematic reviews [16]. To estimate the potential bias in selective reporting, we investigated the association between the sample size and the number of brain loci of significant activation across individual studies [21]. It is expected that studies with a larger sample size would detect more loci than studies with a smaller sample size, and biases may be associated with an over-presentation of the studies with a smaller sample size but a greater number of loci reported [21]. The pattern of a potential bias in reporting and publication was examined by the plot between the sample size and the number of foci across individual studies.

## Results

### Study selection

Based on the search of three electronic databases/indices, 177 records were identified after removing duplicates. Subsequently, 154 records were further screened for their eligibility, and 141 were excluded (see Supplementary Table 1 for the reasons for exclusion). Thirteen studies were eligible to be included in this review (Fig. 1). The studies were categorized into three groups according to the modalities they investigated (Table 2):

1. The first group of studies focused on the interaction between two modalities of oral sensorimotor processing, including somatosensation of food hardness and size [22], somatosensation and chewing movement [23], somatosensation and taste [24], nociception and jaw movement [25], taste and tongue movement [26].
2. The second group focused on the interaction between one modality of oral sensorimotor processing and another non-oral sensory modality, including oral and manual stereognosis [27], taste and olfaction [28, 29], vision and somatosensation [30], somatosensation and audition [31], and audition and mouth actions [32].
3. The third group focused on the interaction between two non-oral sensory modalities, including vision and audition associated with dental anxiety and fear [33], and visual recognition of foods and sensorimotor processing of eating tools [34].

**Table 2:** Study characteristics of the included articles

Group 1: Two modalities of oral sensorimotor processing							
Reference	Participant	Age	Imaging method	Unimodal condition		Bimodal condition (Bi)	Analysis
				Uni1	Uni2		
Narita 2023	7 HA	26.9 ± 6.5	fNIRS	food hardness	food size	n.a.	Uni1 ∩ Uni2
Ishii 2023	9 HA	30.8 ± 8.8	fNIRS	sensorimotor (chewing)	sensory (tongue anesthesia)	chewing with the tongue anesthetized	Modulation of Uni2 on Uni1
Henderson 2016	19 HA, pain-free	34.7 ± 2.4	MRI, ASL	noxious (via injection of hypertonic saline)	sensorimotor (empty chewing)	chewing with continuous infusion of hypertonic saline	Modulation of Uni1 on the association between brain activity of Uni2 and PCS
Hort 2016	12 thermal tasters	30 ± 7	MRI, functional	taste (sweet) at 6 °C	somatosensory (different CO_2_ concentration levels)	sweet samples with different CO_2_ levels	Modulation of Uni2 on Uni1
	12 thermal non-tasters	32 ± 5					
Okamoto 2009	19 HA	32.1 ± 6.9	fNIRS	taste (sugar-based stimuli)	sensorimotor (tongue tapping)	n.a.	Uni1 ∩ Uni2
Group 2: One unimodality of oral sensorimotor processing and one non-oral modality							
Reference	Participant	Age	Imaging method	Unimodal condition		Bimodal condition (Bi)	Analysis
				Uni1	Uni2		
Schumann-Werner 2023	20 HA	20.6–34.6	MRI, functional	oral stereognosis	manual stereognosis	n.a.	Uni1 ∩ Uni2
Suen 2021	35 HA	18–27	MRI, functional	taste (sour)	olfactory (mango)	flavor of sour taste and mango smell	Bi > baseline
Kagawa 2014	11 HA	28.4 ± 5.8	fNIRS	tactile (shape discrimination)	visual (shape memory)	n.a.	Uni1 > Uni2
Eldeghaidy 2011	17 HA^a^	28 ± 8	MRI, functional	taste (sucrose)	olfactory (aroma)	flavour of taste and aroma stimuli	(Bi > Uni1) > (Uni2 > Control)
							(Bi > Uni1) ∩ (Bi > Uni2)
Etzel 2008	16 HA	25–45	MRI, functional	auditory (sounds of mouth/hand actions)	sensorimotor (manipulating a small object with the lips/hands)	n.a.	crossmodal classification
Schulz 2003	10 professional trumpeters	26 ± 2.9	MEG	tactile (via Balloon membranes)	auditory (trumpet tone)	the auditory and tactile stimulation presented simultaneously	Bi > (Uni1 + Uni2)
	9 non-musicians	25 ± 3.9					
Group 3: Two non-oral modalities							
Reference	Participant	Age	Imaging method	Unimodal condition		Bimodal condition (Bi)	Analysis
				Uni1	Uni2		
Yamaguchi 2014	36^b^ HA	23–28	TMS	visual (color photographs of food or non-food)	sensorimotor (holding an eating or non-eating tool)	categorizing the visual stimuli while holding a tool	2 × 2 ANOVA
Hilbert 2014	13 dental phobics	24.92 ± 2.25	MRI, functional	auditory (anxiety arousing drill sounds or neutral sounds)	visual (anxiety arousing dental scenes or neutral scenes	n.a.	Uni1(Anxiety > Neutral) > Uni2 (Anxiety > Neutral)
	13 HA	23.23 ± 3.19					

*fNIRS* functional near-infrared spectroscopy, *HA* healthy adults, *MEG* magnetoencephalography, *MRI* magnetic resonance imaging, *n.a.* not available, *PCS* pain catastrophizing scale, *TMS* transcranial magnetic stimulation.

^a^Only 13 participants were included in the functional MRI analysis. Four were excluded due to excessive head motion.

^b^Only 12 participants were included in Experiment 3.

**Fig. 2:**
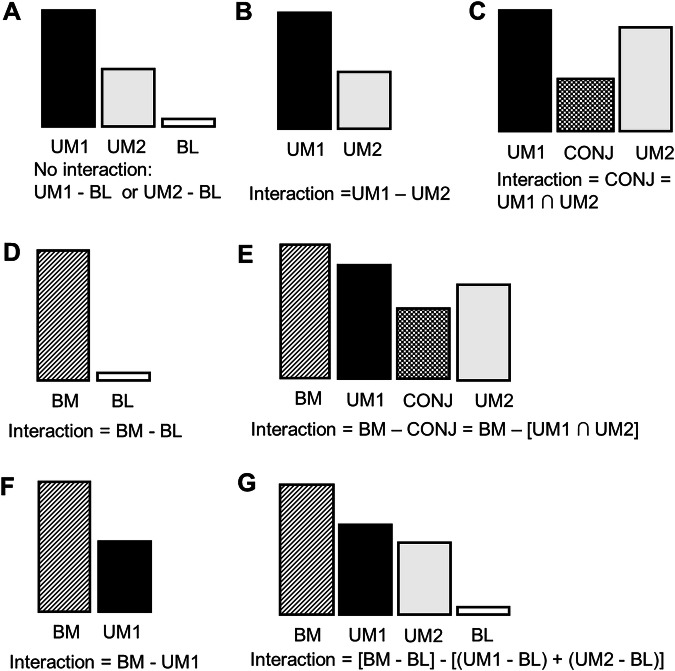
Experimental design of crossmodal interaction. **A** The design investigates the brain features associated with each of the unimodal stimuli (UM1 and UM2) by contrasting them with the baseline (BL) condition. Brain activation from such a design does not reveal the interaction between the two stimuli. **B**, **C** These two designs investigate either the differential pattern of activation between the two unimodal stimuli (i.e., UM1 vs. UM2, panel B) or the common pattern across the two modalities (i.e., UM1 ∩ UM2), **C**. **D**–**G** These four designs include a bimodal condition (BM), i.e., an experimental condition in which UM1 and UM2 are applied concurrently. The BM condition can be contrasted to the baseline condition (i.e., BM vs. BL), **D** or the conjunction of the unimodal conditions (i.e., BM vs. UM1 ∩ UM2, **E**. The interaction can be contrasted by how the effect of the first stimulus (i.e., UM1) is modulated by the additional second stimulus (i.e., BM > UM1, panel F). Finally, the interaction between UM1 and UM2 as well as the main effects of UM1 or UM2 can be contrasted by a 2-by-2 factorial design (**G**).

Some studies, though including task conditions of oral sensorimotor processing, were excluded due to the lack of analysis on crossmodal interaction. A recent study on the association between orofacial pain and visual processing was excluded from this review because only one modality of sensory stimuli (i.e., visual) was investigated [35]. In addition, a study was excluded if it focused on the brain activation of each of individual unimodal stimuli, rather than the interaction between these two stimuli (Fig. 2A).

### Study characteristics

All the studies investigated adults only (Table 2). Three studies included an investigation of different groups of participants, including thermal tasters vs. non-thermal tasters [24], individuals with dental phobic vs. healthy controls [33], and professional musicians vs. non-musicians [31]. In terms of the sensory modalities being investigated, sensorimotor processing associated with kinesthesia and proprioception, including chewing movement [22, 23], jaw movement [25], tongue movement [26], holding eating tools [34], and lip movement [32], as well as somatosensation [22–24, 27, 30, 31] were the most frequently investigated modality. Gustation was investigated in four studies [24, 26, 28, 29]. Vision [30, 33, 34] and audition [31–33] were investigated in three studies, respectively. Olfaction was investigated in two studies [28, 29] and nociception in one study [25] (Table 2). Among the 13 studies, seven used magnetic resonance imaging (MRI) to study the brain features, including six functional MRI studies and a study of arterial spin labeling (ASL). Four studies applied fNIRS. Finally, TMS and MEG were applied in one study, respectively (Table 2). In terms of the analyses of crossmodal interaction, the 13 studies showed a great divergence. In seven studies, the bimodal conditions, i.e., concurrent stimulation from two individual unimodal stimuli, were investigated [23–25, 28, 29, 31, 34].

### Risk of bias in studies

The results of the RoB analysis are summarized in Table 3. In general, all the studies showed a low to moderate degree of RoB, judging from the five items of the RoB assessment (Table 3). The majority of the studies explicitly stated the inclusion and exclusion criteria of the systemic conditions of participants. In contrast, fewer studies investigated the oral conditions of the participants. Most of the functional MRI studies conducted correction of head motion, which is a major factor confounding neuroimaging results. The majority of the studies applied a proper baseline condition for comparison and conducted a correction of multiple comparisons for neuroimaging results.

**Table 3:** Results of risk of bias assessment

Criteria for assessment of risk of bias					
	Systemic condition	Oral function	Motion correction	Task comparison	Threshold
Higher risk	Patients with major systemic diseases were included	The participants with potential deficits in oral sensorimotor function were included	No assessment or correction for head motion was performed	The stimulation task was confounded by baseline factors (no comparison)	Imaging data were not corrected for multiple comparison.
Unclear risk	Not reported	Not reported	Not reported	Not reported	Not reported
Lower risk	Patients with major systemic diseases were excluded	The participants with potential deficits in oral sensorimotor function were excluded	Head motion was examined at the individual basis or using automatic registration	The stimulation task was well controlled by comparing to a baseline condition	Imaging data were corrected for multiple comparison.
Results of assessment of risk of bias					
Narita 2023	2.1	2.1	U	3.2	2.6
Ishii 2023	p.2	p.3	U	p.7	p.6
Schumann-Werner 2023	p.924	U	p.926	p.926	p.927
Suen 2021	p.3	U	p.6	p.3	p.6
Henderson 2016	U^a^	U	p.642	p.641	p.642
Hort 2016	U	U^b^	p.2267	p.2267	p.2267
Hilbert 2014	p.2	U	p.3	p.2	p.3
Kagawa 2014	p.2	U	U	p.2	p.2
Yamaguchi 2014	p.143	U	n.a.	p.144	n.a.
Eldeghaidy 2011	U	U	p.166, p.167	p.165	^c^
Okamoto 2009	U	p.130	p.130	p.130	p.130
Etzel 2008	p.2	U	U	p.2	p.4
Schulz 2003	p.158	U	U	p.158	n.a.

*n.a.* not available, *U* uncertain.

^a^Participants were pain-free.

^b^Taste assessment was conducted.

^c^The results were corrected for multiple comparison in the analysis of main effect but not for the effect of cross-modal interaction.

### Results of individual studies

As shown in Table 2, all the studies have reported statistically significant findings for the crossmodal interaction between the sensory modalities. The findings of brain features are further presented in Table 4. Notably, the studies had different focuses on the sensory modalities, and there existed a great heterogeneity in the experimental design crossmodal interaction. As shown in Fig. 2, the experimental design can be in general categorized into several major types. First, some studies did not include a bimodal condition (BM), only consisting of two experimental conditions for each unimodal stimulus (i.e., UM1 and UM2, Fig. 2B, C). In these studies, crossmodal interaction was referred to as the contrast activation between two unimodal conditions (i.e., UM1-UM2, Fig. 2B) or the conjunction of brain activation between the two conditions (i.e., UM1 ∩ UM2, Fig. 2C). Some studies adopted a BM condition, in which two unimodal stimuli were applied congruently (Fig. 2D–G). Crossmodal interaction was referred to as the contrast between the BM condition and the baseline condition (i.e., no stimuli) (Fig. 2D), the conjunction between UN1 and UN2 (Fig. 2E), or one of the unimodal conditions (Fig. 2F). Finally, some studies adopted a 2-by-2 factorial design to investigate the interaction and main effect of both UM1 and UM2 (Fig. 2G).

**Table 4:** Brain regions associated with crossmodal interaction

Group 1: Two modalities of oral sensorimotor processing															
Study	Modalities		Design	M1	M2	Basal ganglia	Cere-bellum	S1	S2/ RO	PPC	Olfactory	Visual	Insula	CC	PFC
Narita 2023	sensori-motor	somato-sensory	C							R/L^b^		B			
Ishii 2023	sensori-motor	somato-sensory	F							B^c^		B			
Henderson	noxious	sensori-motor	F	L				L					R		R
Hort 2016	gustatory	somato-sensory	F	R	B			B	B	R					
Okamoto 2009	gustatory	sensori-motor	C	B				B							

Group 2: One unimodality of oral sensorimotor processing and one non-oral modality															
Study	Modalities		Design	M1	M2	Basal ganglia	Cere-bellum	S1	S2/ RO	PPC	Olfactory	Visual	Insula	CC	PFC
Schumann-Werner 2023	somato-sensory	somato-sensory	C							L^c^		L			
Suen 2023	gustatory	olfactory	D	R	R	B	B	B	R		L		R	L	R
Kagawa 2014	somato-sensory	visual	B							L		B			
Eldeghaidy 2011	gustatory	olfactory	G		R			R	R	B		R	L	B	B
Etzel 2008	auditory	sensori-motor			B										
Schulz 2003	somato-sensory	auditory	E					B^d^							

Group 3: Two non-oral modalities															
Study	Modalities		Design	M1	M2	Basal ganglia	Cere-bellum	S1	S2/ RO	PPC	Olfactory	Visual	Insula	CC	PFC
Yamaguchi 2014	visual	sensori-motor	G	L											
Hilbert 2014^a^	visual	auditory	B			R							B		L

*CC* anterior/mid-cingulate cortex, *M1* primary motor cortex, *M2* secondary motor cortex, including the supplementary motor area, *PFC* prefrontal cortex, *PPC* posterior parietal cortex, including the superior and inferior parietal lobules and the supramarginal gyrus, *RO* Rolandic area, *S1* primary somatosensory cortex, *S2* secondary somatosensory cortex.

^a^The findings revealed the increased activation in patients with dental phobia compared to healthy controls.

^b^PPC activation was found at the right and the left hemispheres for the food size condition and the food hardness condition, respectively.

**Fig. 3:**
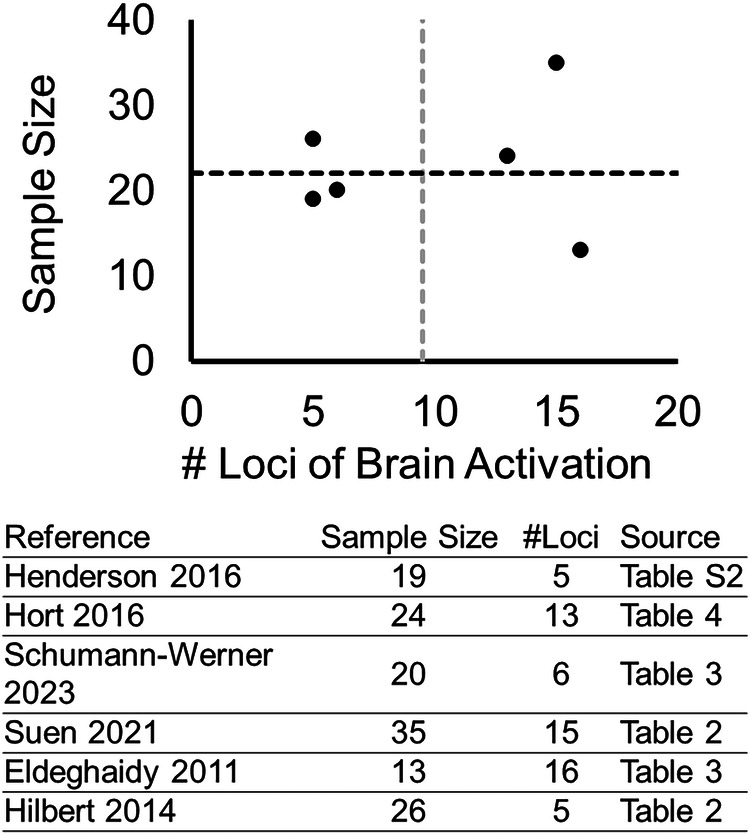
Investigation on potential bias in reporting and publication. Six functional MRI studies that reported the brain coordinates in the Montreal Neurological Institute (MNI) format were selected, and the numbers of loci with significant activation were calculated. The association between the sample size and the number of brain loci across the studies does not show a pronounced pattern of reporting bias (i.e., more publications of small studies with a great number of brain loci reported).

^c^In Fig. 3 only difference at the visual cortex was displayed.

^d^The effect was more pronounced in the trumpeters compared to non-musicians.

As shown in Table 4, the primary motor cortex (M1) and the primary somatosensory cortex (S1) were frequently reported in the studies (Table 4). Notably, activation of these regions was associated with touch or oral sensorimotor processing, including jaw movement [25], tongue movement [26], holding eating tools [34], and somatosensory stimuli [24, 29, 31]. Activation of the secondary motor cortex, including the premotor cortex (PMC) and the supplementary motor area (SMA), was noted in four studies [24, 28, 29, 32]. In contrast, the brain regions associated with crossmodal integration, including the STG, the insula, and the prefrontal cortex (PFC), were not consistently reported in the studies (Table 4). An exception is the posterior parietal cortex (PCC), which was reported in six studies [22–24, 27, 28, 30]. Notably, the pattern of brain activation was associated with the experimental design of individual studies. The study adopting a contrast between BM and the baseline condition (Fig. 2D) showed a more extensive activation [29]. The studies based on fNIRS [22, 23, 26, 30], MEG [31], and TMS [34] also reported a fewer number of brain activation because the investigation was conducted on a limited number of pre-selected brain regions or channels.

### Reporting biases

The results of the assessment of reporting biases are summarized in Fig. 3. Six functional MRI studies that reported the brain coordinates in the Montreal Neurological Institute (MNI) format were selected, and the numbers of loci with significant activation were calculated (see Supplementary Table 2 for detailed coordinates of the brain loci). The sample size and the number of brain loci did not show a pronounced association (i.e., more brain loci reported by smaller studies) that indicates potential bias in reporting and publication (Fig. 3).

## Discussion

### Brain mechanisms associated with oral multisensory processing

A recent neuroimaging meta-analysis of 49 studies revealed that the processing of multi-modal (i.e., auditory, visual, tactile, gustatory, and olfactory) sensory stimuli was associated with brain activation in the temporal lobe, the thalamus, the right insula, and left inferior frontal cortex [8]. Our results on multisensory processing related to oral health, however, did not show such a consistent pattern (Table 4). Here, a more diverse pattern of brain activation was found across the studies. The only brain regions that were repeatedly identified across the studies were the somatosensory area, including S1 and the secondary somatosensory cortex, which were identified in six studies, and the motor area (including M1, PMC, the basal ganglia, and the cerebellum), which were identified in eight studies. Six studies reported the posterior parietal cortex (PPC), which was identified for multisensory integration in previous research [36]. In addition, the brain regions associated with associative learning and attentional control, e.g., the PFC, the insula, the thalamus, and the insula, which were also associated with multisensory integration [37, 38], were not consistently found here (Table 4).

Nevertheless, the current findings disclose a critical pattern that the sensorimotor area may play a key role in oral multisensory processing. This is partly because in most of the included studies, a task condition of oral sensorimotor processing, such as passively perceiving the taste of food or actively moving jaw or tongue, was involved. Notably, the ‘unimodal’ stimulus in the mouth may not induce only a single sensory pathway. For example, when individuals try to perceive the taste of food, tongue movement facilitates the contact between tastants and chemical receptors on the tongue. Therefore, in all the studies including taste stimuli, there was activation in the sensorimotor area [24, 26, 28, 29]. The findings echoed the concept that sensorimotor processing plays a key role in shaping the ‘mouth experience’, i.e., individuals actively explore the intraoral conditions by integrating sensory feedback and motor activity [1].

### A critical evaluation of the experimental design of crossmodal interaction

The review showed great heterogeneity in the experimental design (Table 2). Furthermore, the studies used different approaches to analyze crossmodal interaction, and the divergence of the analytic approach greatly affects how brain features are interpreted. For example, the study by Okamoto et al. analyzed the conjunction of activation between taste and sensorimotor tasks [26]. Because the study investigated only the intersected regions between two task conditions, the brain activation specific to one task (e.g., taste) but not to the other (e.g., tongue tapping) was not shown in the conjunction. The study by Hort et al. investigated the modulational effect of somatosensory stimuli on gustation with three experimental conditions (no, low, and high concentration of CO_2_) [24]. Critically, gustatory stimuli were delivered in all three conditions, and therefore, a contrast between these conditions may not reveal brain activation specific to gustation.

In terms of multisensory integration, the key to ‘integration’ is the interaction between multi-modal stimuli. As shown in Fig. 2A, many neuroimaging studies investigated the effect of two unimodal stimuli, and each of them was contrasted with baseline (control) conditions. However, the findings only revealed the brain features related to each of the unimodal stimuli. The design of Fig. 2B investigated the difference between two unimodal stimuli. The approach may only reveal the brain processing involved in one sensory pathway but not in another. In contrast, the design of Fig. 2C better reflects the common pattern of brain activation by identifying the conjunction of brain regions reported in individual unimodal conditions [17]. The crossmodal interaction may be better contrasted by including a condition of ‘bimodal’ stimulation, i.e., two primary sensory modalities were delivered concurrently. The simplest design is to contrast the brain activation of a bimodal condition to a baseline condition (Fig. 2D). However, the results may only reflect the change of baseline mental activity, such as heightened attention, which is common to both unimodal stimuli and not relevant to crossmodal interaction. To better capture such an interaction, one may investigate the difference between the bimodal condition and the conjunction between two unimodal conditions (Fig. 2E) and identify the effect of interaction using a two-factor design (Fig. 2G). The latter design would better reflect the ‘interaction’ in a statistical sense, i.e., the change of one effect (from the first sensory stimuli) associated with another effect (from the second sensory stimuli) [17]. There are also several studies investigating the modulational effect of one unimodal stimulus on another, as shown in Fig. 2F. The design revealed how one unimodal stimulus was affected by another stimulus.

### Clinical implications

In the current review, all the studies only focused on healthy adults and did not investigate oral multisensory processing related to oral diseases (Table 2). However, the findings disclosed that even in healthy adults, the shaping of multisensory experience related to oral functions is very complicated—it is associated not only with the peripheral structure of the stomatognathic system but also the central nervous system. The findings may have three major impacts on the clinical practice of oral medicine:

1. At present, most of the assessments of oral function focus on motor activity, such as chewing and swallowing function, which plays a key role in clinical assessments [39, 40]. Nevertheless, better performance of sensory assessments, such as oral stereognosis and tactile sensibility, was also associated with masticatory function [41, 42]. In line with this review, the findings suggest that assessments of both motor and sensory abilities, with a focus on the integration of sensorimotor information, should be considered for assessing oral function.
2. Multisensory processing is associated with brain mechanisms, and therefore, individual differences in brain features would play a key role in their multisensory experience of oral functions. This point is especially important to elderly people because a substantial change in brain function and structure is associated with normal aging or diseases. Particularly, a lower cognitive function was associated with not only worse mastication or swallowing but also worse tactile and gustatory function [43, 44]. Further research is required to elucidate the role of multisensory integration in maintaining oral function within patients with cognitive impairment.
3. Recent neuroimaging and animal findings have highlighted the plastic effect on brain structure and function associated with changes in oral function [45, 46]. It is noteworthy that multisensory experience can be sculpted by training [47, 48]. Therefore, in the rehabilitative therapies of oral function (e.g., practicing oral exercises), a potential goal is to enhance individual capacity to integrate multisensory information. In line with this view, recent studies have concluded that in older adults, cortical function plays a key role in the successful training of oral function [45, 46]. Our findings revealed that brain regions other than the sensorimotor cortices may play a key role in the effectiveness of oral rehabilitative training.

### Limitations and further considerations

One should interpret the findings from the current review cautiously with the following limitations. First, the divergent pattern of brain activation associated with oral multisensory processing, as shown in Table 4, reflected a substantial degree of heterogeneity in experimental design. Moreover, six included studies used FNIRS, TMS, and MEG, which were conducted on pre-selected brain regions or channels, rather than a study that investigated the whole brain. Therefore, the study conducted only a qualitative approach, rather than a quantitative analysis (e.g., the coordinate-based meta-analysis), to synthesize neuroimaging findings. Second, as stated in the previous section, there has been no standardized approach to the design of the task conditions for crossmodal interaction. The included studies were not consistent in their experimental design. Half of the studies did not include a condition of “bimodal stimuli”, and only investigated the brain activation when two modalities were delivered separately. Therefore, the brain features revealed by a study should be carefully interpreted according to the experimental design. Third, most of the studies included here were not designed to assess clinical symptoms or treatment effects. In general, the included studies did not present sufficient information on the clinical and behavioral performance of participants. For example, when a study focuses on the association between tactile and sensorimotor processing of chewing, the time to complete a task should be recorded and analyzed, as a critical factor to evaluate the cognitive demands of multisensory integration, and tactile sensibility and masticatory performance should be assessed to evaluate individual baseline capacities. The neuroimaging findings became more difficult to interpret without parallel findings from the clinical and behavioral assessments. Therefore, the findings should not be overdrawn for clinical assessment or diagnosis.

Based on the limitations, we suggested three points to be highlighted in future research on oral multisensory integration. First, the definition of multisensory integration should be clarified and operationalized with a proper experimental design, in which the comparison between bimodal and unimodal conditions plays a key role. Second, clinical and behavioral variables associated with the experimental tasks, which contribute to interpreting neuroimaging findings, should be recorded. Finally, more studies are required to investigate multisensory integration in patients with deficits in oral function.

## Conclusions

The systematic review revealed a complex pattern of brain activation of oral multisensory processing, which can be attributed to the diversity in the experimental design of crossmodal interaction. The findings highlight the role of multisensory integration in maintaining oral health.

## Supplementary information

SupplementaryTable1_PRISMA_checklist

TableS1

TableS2

## Data Availability

The data related to the findings of the study are available in the Supplementary Materials.
