# Supplementary material for: Brain mechanisms of oral multisensory processing related to oral health: a systematic review of neuroimaging findings
Source: BDJ Open. 2025 May 14;11:48. doi: 10.1038/s41405-025-00339-3 (PMC12078481; doi:10.1038/s41405-025-00339-3)
Supplement: Supplementary file 3 — TableS2 [file 41405_2025_339_MOESM3_ESM.docx]

**Table S2.** The number of loci of brain activation from whole-brain analyses

| Study | Number of loci | Source | Contrast | | | | | | |
| --- | --- | --- | --- | --- | --- | --- | --- | --- | --- |
| Henderson 2016 | 5 | Table S2 | increase | | | | | | |
|  |  |  | x | y | z | size | *t* | region | side |
|  |  |  | -48 | 6 | 34 | 32 | 4.19 | M1 | L |
|  |  |  | -38 | -14 | 36 | 43 | 4.8 | S1 | L |
|  |  |  | 10 | 54 | 0 | 25 | 4.45 | MPFC | R |
|  |  |  | Decreased | | | | | | |
|  |  |  | 42 | 0 | 0 | 41 | 4.54 | PINS | R |
|  |  |  | 46 | 20 | 26 | 54 | 4.85 | DLPFC | R |
| Hort 2016 | 13 | Table 4 | thermal taster | | | | | | |
|  |  |  | x | y | z | size | *Z* | region | side |
|  |  |  | -62 | -28 | 16 | 51 | 5.41 | STG | L |
|  |  |  | 64 | -14 | 8 | 26 | 3.34 | STG | R |
|  |  |  | thermal non-taster | | | | | | |
|  |  |  | x | y | z | size | *Z* | region | side |
|  |  |  | 62 | -12 | 42 | 797 | 3.59 | S1 | R |
|  |  |  | 54 | -16 | 22 |  | 3.25 | S2/RO | R |
|  |  |  | 58 | -20 | 26 |  | 3.23 | SMG | R |
|  |  |  | -52 | -14 | 46 |  | 3.52 | S1 | L |
|  |  |  | 62 | -26 | 18 |  | 4.55 | SMG | R |
|  |  |  | 68 | -28 | 8 |  | 3.89 | STG | R |
|  |  |  | 56 | 0 | 6 |  | 4.55 | S2/RO | R |
|  |  |  | -56 | 2 | 14 | 44 | 3.54 | S2/RO | L |
|  |  |  | 2 | 0 | 50 | 312 | 3.31 | SMA | R |
|  |  |  | -6 | -10 | 48 |  | 3.34 | SMA | L |
|  |  |  | 60 | -2 | 42 |  | 3.58 | M1 | R |
| Schumann-Werner 2023 | 6 | Table 3 | conjunction | | | | | | |
|  |  |  | x | y | z | size | *Z* | region | side |
|  |  |  | -40 | -60 | -6 | n/a | 5.01 | IOG | L |
|  |  |  | -50 | -66 | -2 |  | 4.75 | MTG | L |
|  |  |  | -50 | -68 | -6 |  | 4.71 | ITG | L |
|  |  |  | -42 | -30 | 40 |  | 4.43 | IPL | L |
|  |  |  | -48 | -26 | 36 |  | 3.87 | IPL | L |
|  |  |  | -54 | -30 | 42 |  | 3.59 | IPL | L |
| Suen 2023 | 15 | Table 2 | taste plus smell | | | | | | |
|  |  |  | x | y | z | size | *t* | region | side |
|  |  |  | 48 | -10 | 34 | 2331 | 7.05 | S1 | R |
|  |  |  | 60 | 2 | 28 |  | 6.42 | M1 | R |
|  |  |  | 12 | 2 | 1 |  | 6.29 | PAL | R |
|  |  |  | -12 | 2 | -2 |  | 5.90 | PAL | L |
|  |  |  | -9 | -1 | -8 |  | 5.42 | OLF | L |
|  |  |  | 6 | 2 | 64 |  | 5.12 | SMA | R |
|  |  |  | 33 | 14 | 13 |  | 4.95 | AINS | R |
|  |  |  | 39 | -4 | 19 |  | 4.70 | S2/RO | R |
|  |  |  | 39 | -1 | 4 |  | 3.92 | MINS | R |
|  |  |  | 12 | -58 | -23 | 425 | 6.82 | CB | R |
|  |  |  | -12 | -61 | -23 |  | 6.34 | CB | L |
|  |  |  | -48 | -16 | 31 | 310 | 6.26 | S1 | L |
|  |  |  | -57 | -13 | 28 |  | 6.06 | S1 | L |
|  |  |  | -3 | -25 | 28 | 190 | 5.55 | MCC | L |
|  |  |  | 33 | 41 | 4 | 127 | 5.28 | MFG | R |
| Eldeghaidy 2011 | 16 | Table 3 | congruent flavour stimuli (subtraction analysis) | | | | | | |
|  |  |  | x | y | z | size | *Z* | region | side |
|  |  |  | 10 | 40 | 20 | n/a | 2.37 | ACC | R |
|  |  |  | 10 | 46 | 28 |  | 2.34 | ACC | R |
|  |  |  | 10 | 0 | 50 |  | 2.34 | SMA | R |
|  |  |  | -34 | 10 | -2 |  | 2.39 | AINS | L |
|  |  |  | -48 | 10 | -6 |  | 2.43 | STG | L |
|  |  |  | 58 | 10 | 18 |  | 2.33 | DLPFC | R |
|  |  |  | -4 | 42 | -16 |  | 2.46 | VMPFC | L |
|  |  |  | -12 | 38 | -6 |  | 2.45 | ACC | L |
|  |  |  | 30 | 2 | -18 |  | 2.39 | AMYG | R |
|  |  |  | 40 | -44 | -12 |  | 3.93 | FUS | R |
|  |  |  | -44 | -44 | -12 |  | 2.53 | ITG | L |
|  |  |  | 62 | -26 | 32 |  | 2.42 | SMG | R |
|  |  |  | -60 | -20 | 42 |  | 2.41 | SMG | L |
|  |  |  | 54 | -6 | 26 |  | 3.08 | S1 | R |
|  |  |  | -62 | -46 | 18 |  | 2.38 | STG | L |
|  |  |  | -62 | -40 | 8 |  | 2.33 | MTG | L |
|  | 9 | Table 4 | congruent flavour stimuli (conjugate analysis) | | | | | | |
|  |  |  | x | y | z | size | *Z* | region | side |
|  |  |  | -56 | -26 | 40 |  | 2.33 | SMG | L |
|  |  |  | 48 | 0 | 10 |  | 2.68 | S2/RO | R |
|  |  |  | -6 | 32 | -6 |  | 2.38 | ACC | L |
|  |  |  | 8 | -48 | 24 |  | 2.34 | PCC | R |
|  |  |  | -12 | -42 | 32 |  | 2.33 | PCC | L |
|  |  |  | -8 | 46 | -14 |  | 2.49 | VMPFC | L |
|  |  |  | -14 | 38 | -12 |  | 2.72 | VMPFC | L |
|  |  |  | 62 | -28 | 30 |  | 2.71 | SMG | R |
|  |  |  | -60 | -32 | 38 |  | 2.46 | SMG | L |
| Hilbert 2014 | 5 | Table 2 | auditory vs. visual, between group | | | | | | |
|  |  |  | x | y | z | size | *F* | region | side |
|  |  |  | -32 | 14 | -16 | 326 | 4.93 | AINS | L |
|  |  |  | 48 | 6 | -6 | 165 | 4.44 | AINS | R |
|  |  |  | -12 | 50 | -6 | 382 | 4.92 | OFC | L |
|  |  |  | -14 | -58 | 40 | 64 | 3.69 | PCUN | L |
|  |  |  | visual vs. auditory, between group | | | | | | |
|  |  |  | x | y | z | size | *F* | region | side |
|  |  |  | -28 | -6 | 24 | 157 | 5.05 | CAU | R |
